# Periparturient stress and immune suppression as a potential cause of retained placenta in highly productive dairy cows: examples of prevention

**DOI:** 10.1186/s13028-015-0175-2

**Published:** 2015-12-02

**Authors:** Ryszard Mordak, Stewart Peter Anthony

**Affiliations:** Department of Internal Medicine and Clinic of Diseases of Horses, Dogs and Cats, Faculty of Veterinary Medicine, Wrocław University of Environmental and Life Sciences, Grunwaldzki Sq. 47, 50-366 Wrocław, Poland; Veterinary Clinic Stewart and Partners, 2 Brooke Street, Dudley, DY2 8RB UK

**Keywords:** Cows, Periparturient stress, Retained placenta, Prevention

## Abstract

The immune system during the periparturient period is impaired. At this time the most important factor causing immune-suppression in highly productive cows is metabolic stress resulting from hormonal and metabolic fluctuations, a negative energy balance, shortage of proteins, minerals and vitamins which are required to meet the demands of the fetus as well as the onset of lactation. This stress can activate the hypothalamic-pituitary-adrenocortical axis (HPA), which results in increase plasma corticosteroids. As a result, the cortisol concentration during the periparturient period increases by several folds particularly on the day of calving. Cortisol is a powerful immune-suppressive agent. During stress, this hormone causes depression of the leukocyte proliferation and their functions. Decreased phagocytosis of neutrophils, decreased cytotoxic ability of lymphocytes, as well as depressed activity of their cytokines, make it impossible for the normal, efficient maternal immune recognition and rejection of fetal membranes (as a foreign, allogeneic tissue expressed fetal antigens—MHC class I proteins by trophoblast cells) and finally results in their retention in cows. The metabolic periparturient stress also activates production of catecholamines, especially adrenalin. Adrenalin activates adrenoreceptors of the myometrium and then causes hypotony or atony of the uterus. Thus, cortisol and adrenalin inhibit rejection and expulsion of fetal membranes and cause their retention. These mechanisms of retained placenta (RP) often have a metabolic etiology and occur in herds, where important infectious diseases causing placentitis are absent or prevented. The aim of this article is to show the fundamental mechanisms occurring during periparturient stress and the accompanied immune-suppression in cows, as well as their consequences in relation to RP. The paper also gives examples of the symptomatic prevention of RP in cows caused by metabolic and immune suppressive factors. The prevention of RP was carried out using drugs which inhibit the activity of cortisol or adrenalin in dairy cows during calving.

## Background

Current knowledge of physiology and immunology shows that the periparturient period in highly productive dairy cows is a critical period for their metabolism, immunity, health and further fertility [1–4]. The immune system is depressed in cows around the time of parturition [5]. During this period the most important factor causing impairment of the immune system in high yielding cows is periparturient stress caused by hormonal and metabolic fluctuations, especially, a negative energy balance, shortage of proteins, minerals and vitamins associated with the demands of a mature fetus, as well as the onset of lactation [3, 6]. This metabolic stress stimulates corticotrophic releasing hormone (CRH). In consequence, adrenocorticotrophic releasing hormone (ACTH) is produced. Then, ACTH activates the hypothalamic-pituitary axis (HPA) and increases plasma corticosteroids, even during physiological activation at calving time [7]. According to cited authors, the mean baseline cortisol levels in cows during normal conditions are near 5 ng/ml, but they may range from 10 to around 20 ng/ml, but during controlled stress, the plasma cortisol concentration increases usually 20–30 %. Cows during parturition may exhibit a 3–4 folds increase of baseline plasma cortisol concentration, but during metabolic disorders, especially in hypocalcemic cows at calving time may observe a 5–7 increase of serum cortisol level [8]. Also other factors present at calving like dystocia and even veterinary aid act as a stressors and in consequence cause an elevated level of cortisol and immune suppression in cows [9]. The periparturient stress also may activate production of catecholamines, especially adrenalin, which during stimulation of myometrial adrenoceptors causes hypotony or atony of the uterus. Immunosuppression and hypomotility of uterus in cows are very significant, because they may be dangerous from the point of view parturition–expulsion of the fetus and placenta in cows.

### Search strategy

This review is based on a search in PubMed (http://www.ncbi.nlm.nih.gov/pubmed) using the terms “cows, retained placenta, periparturient/transition period, periparturient stress, prevention” combined with our own bibliography sources, especially Cattle Practice Journal, which was received individually by post from British Cattle Veterinary Association during last 10 years and other sources also using The University Library and our experiences with clinical works, which were used to compare and critically evaluate the literature.

## Review

### Important mechanisms, which may act during periparturient period in relation to retained placenta in cows

During the periparturient period and in conditions of immune suppression, the proliferation of leukocytes (especially lymphocytes and neutrophils) is severely depressed. Then also the fundamental functions of these cells, such as ability to aggregate and phagocytosis of neutrophils, cytotoxic activity of lymphocytes, as well as production of chemotactic cytokine IL-8 activating these leukocytesare usually reduced at this time [10]. Due to these changes, the normal efficient maternal immune response and expulsion of fetal membranes is impaired, resulting in their retention in cows [11].

During normal parturition, maternal immunological recognition of fetal antigens (MHC class I proteins) are expressed by trophoblast cells, triggers triggering an immune-inflammatory response, which leads to effective separation and rejection of the fetal placenta (as a foreign tissue, similar to an allogeneic transplant) and contributes to the expulsion of the fetal membranes within 12–24 h after calving [12, 13]. In addition, during periparturient stress, cortisol also decreases the ability of expression of major histocompatibility complex (MHC) molecules and production of prostaglandins. The unbalanced concentrations of prostaglandin PGF2α and prostacyclins were observed in cows with RP, in which the mean levels of these hormones in peripheral blood plasma were significantly lower than that in cows with normal calving [14].

In herds of high yielding cows which are at low risk from infectious diseases (which may cause retention of fetal membranes), the most important pathogenic factor causing RP is immune suppression during parturition. Immune suppression of around 20–30 % is serious, but under current farm conditions is considered “normal” in cows with a milk productivity of 8000 liters per lactation period [5]. In these conditions the clinical cases of RP are not often observed, and they usually include several percent of cows in the herd. In more complicated cases where the level of immune-suppression is severe, RP may be observed in above 10 % cows of herd. The incidence of RP can also be a marker of a cow’s immunity around parturition in herds where the problem of infectious diseases is not present. During the periparturient period—critical time, essentially all dairy cows experience, reduced feed intake, negative energy balance, insulin resistance, hypocalcemia, reduced immune function, as well as bacterial contamination of the uterus soon before or after calving. The different methods of early detection include systemic monitoring of health, level of milk production, feed intake, body condition and simple metabolic tests ought to be performed on farms [15]. The cited author shows the importance of monitored herd data and simple measurements like, prepartum increased concentration of non-esterified fatty acids (NEFA), especially when their level above 0.4 mmol/l in7–10 days before expected calving is associated with increased risk of RP, as well as other health problems after parturition like displaced abomasum (DA) and other losses in production. Thus, the periparturient metabolic disorders, stress and the accompanying suppression of immune response in dairy cows may be assessed by different methods during clinical and laboratory examinations. Monitoring of the production and health in herd, by the use of simple biochemical and homological profiles of blood and milk performance in the representative group of cows in technological groups of around 1 week before and after calving [2, 3, 16, 17], or by the use of more specialized laboratory methods like measurement of leukocyte functions, plasma interleukin IL-8 concentration, or calcium content in peripheral blood mononuclear cells [8, 10, 11, 18].

Current knowledge of pathogenesis of placental retention in high yielding cows is that it depends of their efficient immunity [12, 19]. The authors showed that in the immunological mechanism of RP there is a significant role for the chemotactic factor IL-8 of leucocytes, which are found in the placentomes of cows which have had a normal placental separation, however is absent in placentomes tested from cows with RP. In addition, blood leucocytes, especially neutrophils of cows with RP are less reactive to chemotactic stimuli than in cows with normal placental rejection. This has also been shown by other research workers [10] where at calving plasma IL-8 (chemotactic cytokine for neutrophils) concentration was observed to be lower in cows with RP (average 51 pg/ml) than in cows expelling the placenta normally (on average more than two and half times higher 134 pg/ml). The results obtained by the authors suggest that neutrophil function is a determining factor for the development of RP in dairy cows. The authors also suggested that depressed production of chemotactic cytokine IL-8 may be a factor affecting neutrophil function in cows in the development of placental retention [11]. In that research, the examined neutrophil function was the ability of neutrophils to recognize fetal (foreign) cotyledon tissue, as measured by the chemotaxis assay as well as neutrophil killing ability, which was estimated by determining myeloperoxidase activity. In the bovine RP, reduction of phagocytic activity of macrophages is also noted [20]. Results of this research indicate that there are functional differences in placental macrophages between a normal and retained placenta. Neutrophil mieloperoxidase was reduced in periparturient cows, especially during subclinical or clinical milk fever [19]. The association between neutrophil function and periparturient disorders in cows has been confirmed by other research workers [21].

Characteristic bovine cotyledonary synepitheliochorial structure of the placenta, is its evolution with real different conditions during pregnancy and during parturition or abortion may have significant influence on incidence of RP in cows [22]. Placental maturation is especially important for immunological recognition and rejection of fetal membranes (trophoblast), with the occurrence of the required number of binucleate cells of the trophoblast and flattening of endometrial cells shortly before the normal parturition in cows [23, 24]. In most species of mammals as well as in cattle, trophoblast cells (with their binucleate cells) do not express classical MHC class I molecules and a lack of these antigens is believed to protect those cells and, as a consequence protects the placenta and fetus from attack by the maternal immune system [12, 25]. The binucleate cells have various hormonal (production of steroids, placental lactogen) and maintenance functions for the placenta. Binucleate cells can also migrate across the interface and fuse with endometrial epithelial cells forming trinucleate—hybrid cells as a fetomaternal syncytium [26]. After fusion hybrid, endometrial-trophoblast cells are unable to express maternal MHC class I molecules because, especially in cattle, endometrial cell class I antigen expression is shut down [27]. In other species, presentation of fetal antigens by maternal MHC class I molecules could probably provoke an attack by maternal cytotoxic Tlymphocytes on trophoblast cells through direct recognition of their antigens, however in cattle a lack of MHC class I expression by cryptal endometrial epithelial cells protects the fetus from immune mediated rejection [12]. Independently, fetal-trophoblast MHC class I peptides originating from destructed trophoblast cellsduring calving or abortion can be represented indirectly by specialist maternal antigen presenting cells (APC) exactly by MHC class II molecules (antigens) of dendritic cells or macrophages and can be recognized by helper (h1) Tlymphocytes [13]. According to cited authors, the recognition of trophoblast antigens by helper (h1) Tlymphocytes is transferred by cytokines (IL-2) on cytotoxic Tlymphocytes for activation of apoptosis, as a cellular response. In addition, independently acting helper (h2) T lymphocytes recognize trophoblast antigens through dendritic cells or macrophages and stimulate the activation of B lymphocytes as a humoral response. As a consequence activated lymphocytes and APC produce IL-8, which attract neutrophils to the chemotactic site on placentomes and activate phagocytocis. Neutrophils can be also independently recruited and activated by the components C3 and C5 as an effective immunological response against various antigens like trophoblast during parturition or like pathogens during infection [28]. Each of the activated leukocytes and their cytokines cause a typical immune-inflammatory reaction on allogeneic trophoblast tissue, adequately to the maturation of the placenta and the level of maternal immunity [29]. The results of other newer research show, that reduced levels of pro-inflammatory cytokines (especially IL-6) assessed in conditions of stimulation of leukocytes in periparturient dairy cows using ex vivo whole blood stimulation assay with lipopolysaccharides—LPS (−3 to 3 days from parturition) were observed only after calving [30]. Moreover, according to cited authors, the release of higher levels of IL-6 in the transition period, with low LPS dose, suggests crucial role of this cytokine in the regulation of inflammatory response around calving. Cases of RP observed after expulsion of fetus in cows usually are considered as an unfinished and complicated calving.

The importance of the placental maturation and the role of immune rejection corroborates other research work, where the number of the placentomal binucleate cells and apoptotic bodies (as an effect of cytotoxic T lymphocytes activity) was lower in cows with induced parturition with dexamethasone than in cows calved at normal term [31]. RP may be associated with oxidative stress causing damage of the important intracellular placentomal structures, like DNA and other [32], and resulting in the appearance of the increased synthesis of poly(ADP-ribose) polymerase, which was detected in all tissues of placetomes in cows with retention of fetal membranes [33].

### Retained placenta in cows—clinical and laboratory aspects

Retention of fetal membranes in cows is a very serious disorder which occurs in the last phase of parturition. It has a significant negative influence on health, welfare, milk productivity and further reproduction in the postpartum period [34]. There are numerous links between metabolic disorders, inflammation, immune function, RP and further consequences as a reproductive tract disease, which often occurs several weeks after calving [3]. Within 6–9 h of delivery, the fetal membranes which remain in the uterus begin to deteriorate and become a suitable culture medium for various bacteria. These bacteria initially cause an acute puerperal metritis and later a clinical or subclinical endometritis, which results in the late involution of the uterus and the late regression of the ovarian corpus luteum after calving. Cumulatively 35–50 % of cows have at least one form of pathological reproductive tract inflammation diagnosed during clinical or cytological examinations between 3 and 7 weeks postpartum [35]. According to the cited author, an excessive pro-inflammatory state early in postpartum period (especially during RP) appears to be a key feature of cows with endometritis approximately 1 month later. Consequently this leads to other health problems such as mastitis, reproductive complications and infertility [36]. During acute puerperal metritis in cows, measured on third day after expulsion of the fetus, an increased level of acute phase proteins (APP) in the blood is noted, involving haptoglobin, the serum concentration of which reaches near 3 g/l in one third of cases of RP (mean 2.48 g/l) [37]. Haptoglobin may be a marker for monitoring the calving time, when in normal conditions without RP its levels were observed at around 0.5–0.7 g/l [38]. Haptoglobin was shown also a good parameter for assessment of acute postpartum metritis and endometritis in cows, where in severe case its level reach values above 0.7 g/l [39].

Prevention of the periparturient period health problems and RP as a multifactorial case in dairy cows involves usually clinical and laboratory monitoring with the use biochemical or hematological profiles of the blood performed in representative number of animals shortly before and after calving as well as during lactation period [2, 16, 17, 40]. Different productive and metabolic disorders may be observed before, during or after calving as factors of risk, etiology of RP or consequences induced by retention of fetal memebranes in dairy cows [41]. Cows at risk of metabolic disorders and RP may be diagnosed by the use different metabolic profiles involving increased level of lipid metabolites, NEFA, beta-hydroxybutyrates (BHB), cholesterol, decreased levels of vitamin A, vitamin E, total antioxidants and paraoxonase (PON) which as a liver protein released to the blood stream may be a marker of the liver function and indicates their chronic damage by reduced value [42]. In cited research mean value of PON 66.4 U/ml measured at and after calving was drastically reduced in chronic liver damage and was negatively correlated with haptoglobin. In cows with RP,not only hypocalcemiais observed but also reduced serum zinc concentration, increased aspartate transaminase (AST) activity and neutropenia are noted [43]. According to the cited authors especially neutropenia seems to be a co-factor involved pathogenesis of RP in dairy cows. Often the clinical consequences of RP in cows are so serious that they require systemic diagnostic tests for reproductivetract infections and inflammations [44]. Sometimes the real histeroscopic picture of postparturient uterus in cows can also be a good diagnostic tool in veterinary practice [45].

From the farmers point of view, every case of RP in cows is associated with significant financial losses resulting from the cost of therapy, decreased milk yield, decreased body condition, decreased fertility and higher culling rates. The fact that serious health problems occur on almost all dairy farms to varying degrees, depends on the standard of nutrition and husbandry of the individual herds. Fetal membrane retention is a multifactorial health problem, which mainly has an infectious or metabolic origin as well as other reasons and may also be associated with psychological factors that as stressors, are able to activate the HPA and impair immune response [46]. Infectious bacterial or viral factors often cause placentitis during pregnancy which often results in abortion or premature birth when the placenta has not matured [23]. An immature placenta cannot be normally rejected by maternal immune system. In addition, during placentitis a pathological, strong connection between tissues in placentomes is observed specially between fetal membranes and the uterus. Currently the number of these cases is not significant on modern farms because most serious infectious diseases such as brucellosis or other communicable diseases are effectively eliminated or prevented by official monitoring or vaccination programs. On modern dairy farms with high yielding dairy cows, parturition often occurs at term when the placenta is mature but RP occurs due to nutritional, metabolic and immune suppressive factors rather than infectious causes. RP is noted in almost 8 % of the population of dairy cows in the USA according to National Animal Health Monitor System [6], around 10 % dairy cows in EU [47] and it is usually the third most important health problem behind mastitis and lameness.

### Retained placenta in cows—examples of prevention

The most commonly used methods for the prevention of metabolic disorders in cows are all the methods farmers use to eliminate and minimize various potential etiological causes. Ensuring an adequate balanced diet (energy and minerals) in the dry period and nutritional prophylaxis around the time calving, as well as clinical and laboratory monitoring of herds have a fundamental role. These preventative measures can reveal but often cannot eliminate metabolic health problems as well as RP. Apart from nutritional management in problematic herds of dairy Holstein–Friesian cows, other strategies can be employed to prevent RP, such as the improvement of animal welfare, hygiene during calving, reducing stress, infections, idiopathic factors, hormonal imbalance as well as veterinary support of parturition by the use of prostaglandin PGF2αor oxytocin [48, 49]. However, another effective methods appears to be using lysozyme dimer—immune modulator before calving [50, 51] *or administering during calving immediately* after expulsion of the fetus [52]. This appears to be the optimal time for administration of lysozyme dimer in order to support immune rejection of fetal membranes in cows. Lysozyme dimer stimulates the production of interleukins: IL-1, IL- 2, IL-6, and other cytokines like interferon (INF-γ) and tumor necrosis factor (TNF-α) [53] and activates the proliferation of various types of leukocytes also neutropliles and lymphocytes [54]. Lysozyme dimer modulates the synthesis and secretion of TNF-α in varied species of animals and it activates in vivo phagocytosis of granulocytes and macrophages as well as protecting against antibiotic immune suppression [55].

All the properties of lysozyme dimer are required for the effective maternal immune-inflammatory response to occur during recognition and rejection of the fetal placenta. All these immune stimulating activities of lysozyme dimer are in contrast to the immune-suppressive action of cortisol, which is present in higher serum concentrations during metabolic stress at parturition. The action of lysozyme dimer can cancel out almost all of the immune suppressive effects of corticosteroids and supports the rejection of fetal membranes during calving. The use of lysozyme dimer however is symptomatic and does not eliminate the primary cases. The newer research shows that in the periparturient period the lower levels of serum lisozyme concentration in cows (around 1.3 µg/ml) observed especially in tested high-yielding dairy animals with low Liver Functionality Index—experiencing hepatic and other health problems correlated with greater IL-6 levels and with higher ceruloplasmin concentrations [38]. The cited authors observed also in vitro model test, the significant relationship among higher lysozyme concentrations above >4.5 µg/ml and increased spontaneous secretion of IL-8 cytokine which have a positive influence on chemotaxis of neutophils and lymphocytes. In my own observations the incidence of RP in cows, which received immune-support, by the use of one intramuscular injection of lysozyme dimer at a dose rate of 0.01 g/kg bwt just after calving was nearly three times lower in comparison to cows which had not received this immune support just after expulsion of fetus [52]. The percentage of cows with RP earlier supported immunologically during calving was significantly lower especially in problematic herds in cows in which parturitions were resolved with manual intervention (an average about 16 % in group of cows without support and an average about 5 % in group of cows with lysozyme dimer support). The results of clinical observations were later confirmed in a larger population of cows about 200 animals in several problematic farms (data not shown).Immune modulation by the use lysozyme dimer during calving, was shown to be very effective for dairy cows in problematic herds especially, with a high incidence of RP, where other methods of preventions were not effective. The use of immune modulation for the prevention of RP has been shown to be a profitable method of prevention in cows, particularly in those where manual intervention during delivery was necessary. It is therefore likely that accepted calving aids, such as manual manipulation of the fetus and traction using ropes or chains during calving might cause an additional stress and have a significant negative influence on the expulsion of fetal membranes.

Current knowledge indicates the importance of a maternal immune system, which most efficiently recognize and reject the fetal placenta as a foreign tissue, similar to an allogeneic transplant, as well as an immune—inflammatory reaction during parturition. When used during calving, the immune modulator supports the repair and stimulation of the depressed immune system. This is very important from the point of view of the metabolic pathogenesis of RP in cows which have their parturition in normal term. This method may be the vehicle for immunological support at the critical time of parturition in high yielding cows with mature placenta. This immunological support is also administered as a therapy for mastitis in cows as well as various other infections of the digestive, respiratory and reproductive system in animals [55]. In these cases, support of immune system is very important and necessary for effects of therapy especially as infections require antibiotics, which kill bacteria. However, antibiotics used during therapy decrease the functions of the immune system.

Symptomatic prophylaxis of RP caused by metabolic stress in dairy cows may be implemented during calving not only using drugs, which suppress carazolol activity, but also by the use of drugs that reduce the effect of adrenalin, which have negative influence on uterine motility. Beta blockers can be used to protect the uterus from atony or hypotony caused by increased production of adrenalin due to stress. Beta blockers are effective in blocking myometrial adrenoreceptors during prevention and therapy of RP caused by stress of difficult birth in cows, as well as during therapy of post-partum metritis and delayed uterine involution also in combination with other drugs like PGF2α and antibiotics [56]. In cited study performed in highly problematic herds of cows, the effects of beta receptor blocker(carazolol) given just after parturition in dose 5 or 0.5 mg cloprostenol as a prevention of RP were effective because these cases occurred by the rate of 14.2 % in carazolol group, around 38 % in cloprostenol group and around 55 % in control group without prevention. The method of maintaining normal uterine motility with the use of one intramuscular injection of propranolol—a beta blocker at a dose of 50 mg per cow during parturition, was shown to be effective [57]. In cited observations on several farms, the number of cows treated with propranolol, just after expulsion of the fetus and presenting with RP, was significantly lower (nearly two and half times lower) in comparison to cows which had not received this blockade of beta adrenoreceptors just after expulsion of fetus (5.2 % in propranolol group and 12.7 % in control group).

## Conclusions

In the periparturient period highly productive dairy cows exhibit significant risk of occurrence of metabolic disorders, immune dysfunctions, and different health problems. Metabolic disorders have a nutritional basis and they occur often in periparturient cows as a result of a negative energy balance and a lack of minerals and vitamins [3, 58]. In addition, these metabolic disorders cause periparturient stress, which may result in defective separation of fetal membranes and in consequence their retention during calving [59]. The nutritional and metabolic causes of RP are very difficult to eliminate on modern dairy farms. In perspective, cows constantly need to optimize the nutritional and environmental conditions, as well as other methods for the effective support of the immune system during the periparturient period in cows. Managing transition period health for reproductive performance in dairy cows, as well as specific prevention and monitoring of the herds in relation to metabolic disorders (negative energy balance, ketosis or hypocalcaemia) and depressed immune functions are essential on modern farms [4]. Systemic, periodical diagnostic monitoring using blood and milk tests in representative groups of cows often may be insufficient for detection of many important, especially subclinical disorders. Modern practice needs constant daily monitoring of the health in dairy cows for controlling their milk production and different potential risks, especially in the critical transition period. Daily testing of blood parameters on one drop of blood or daily testing of milk parameters, using new laser techniques Vis/NIS (Visible Near Infrared Spectroscopy) seems to be a good solution today and in the future for a current “on line” and “in line” monitoring of the metabolic status of cows and their milk gland [60]. This technique is very useful and low input for farmers in the supervision of the health in milking cows, as well as it allows for rapid response to occurring metabolic disorders [61]. The current, necessary systemic monitoring of dairy herds requires the use of a wider range of diagnosis, according to the health problems, or risks but it may be performed in relation to the interest and financial capacity of farmers.

## Abbreviations

APC: antigen presenting cells
APP: acute phase proteins
BHB: beta-hydroxybutyrates
HPA: hypothalamic-pituitary-adrenocortical axis
IL: interleukin
LPS: lipopolysaccharides
MHC: major histocompatibility complex
NEFA: non-esterified fatty acids
PON: paraoxonase
RP: retained placenta
TNF-α: tumor necrosis factor alpha
