# Erratum to: Periparturient stress and immune suppression as a potential cause of retained placenta in highly productive dairy cows: examples of prevention

**DOI:** 10.1186/s13028-015-0180-5

**Published:** 2016-01-08

**Authors:** Ryszard Mordak, Peter Anthony Stewart

**Affiliations:** 1 Department of Internal Medicine and Clinic of Diseases of Horses, Dogs and Cats, Faculty of Veterinary Medicine, Wrocław University of Environmental and Life Sciences, Grunwaldzki Sq. 47, 50-366 Wrocław, Poland; 2 Veterinary Clinic Stewart and Partners, 2 Brooke Street, Dudley, DY2 8RB UK

## Erratum to: Acta Vet Scand (2015) 57:84 DOI 10.1186/s13028-015-0175-2

There was an error in the final printing of one of the author’s names in the original article [1]. It was printed as “Stewart Peter Anthony” but should be written as “Peter Anthony Stewart” instead where Stewart is the surname and not the first name. The copyright details were also wrong, “© 2015 Mordak and Anthony”, as a consequence of this. The correct copyright details are: “© 2015 Mordak and Stewart”.
